# A Review on the Effects of ZnO Nanowire Morphology on the Performance of Interpenetrating Bulk Heterojunction Quantum Dot Solar Cells

**DOI:** 10.3390/nano12010114

**Published:** 2021-12-30

**Authors:** Meibo Xing, Longxiang Wang, Ruixiang Wang

**Affiliations:** Beijing Engineering Research Centre of Sustainable Energy and Buildings, School of Environment and Energy Engineering, Beijing University of Civil Engineering and Architecture, Beijing 100044, China; xingmeibo@bucea.edu.cn (M.X.); wlx8611@163.com (L.W.)

**Keywords:** interpenetrating bulk heterojunction, quantum dot solar cells, ZnO nanowire, synthetic method, geometric optimization, defect passivation

## Abstract

Interpenetrating bulk heterojunction (IBHJ) quantum dot solar cells (QDSCs) offer a direct pathway for electrical contacts to overcome the trade-off between light absorption and carrier extraction. However, their complex three-dimensional structure creates higher requirements for the optimization of their design due to their more difficult interface defect states control, more complex light capture mechanism, and more advanced QD deposition technology. ZnO nanowire (NW) has been widely used as the electron transport layer (ETL) for this structure. Hence, the optimization of the ZnO NW morphology (such as density, length, and surface defects) is the key to improving the photoelectric performance of these SCs. In this study, the morphology control principles of ZnO NW for different synthetic methods are discussed. Furthermore, the effects of the density and length of the NW on the collection of photocarriers and their light capture effects are investigated. It is indicated that the NW spacing determines the transverse collection of electrons, while the length of the NW and the thickness of the SC often affect the longitudinal collection of holes. Finally, the optimization strategies for the geometrical morphology of and defect passivation in ZnO NWs are proposed to improve the efficiency of IBHJ QDSCs.

## 1. Introduction

The world environmental and energy crisis calls for the imminent development of renewable or nonconventional energies. As the most abundant source of clean energy on the planet, solar energy is an important approach to achieving the goal of carbon neutrality in the future [1]. To date, three generations of solar cells have been evolved to harvest solar energy as efficiently as possible [2], but they are still fundamentally limited in terms of solar cell efficiency, including thermalization losses from the absorption of high-energy photons, the non-absorption of low-energy photons, extraction losses due to unavoidable charge carrier recombination, and other mechanisms of energy loss [3].

Quantum dot solar cells offer the advantage of a zero-dimensional structure [4]. Due to their remarkable multiple exciton generation [5,6], hot carrier effect [7], possible intermediate band cell structure [8,9,10], and multi-junction cell structure [11,12], QDSCs offer the real possibility of boosting energy conversion efficiency beyond the Schockley and Queisser (SQ) limit of 32% for traditional silicon-based SCs [13]. In the past few decades, QDs have been widely researched and integrated in various kinds of SCs, such as Schottky junction colloidal quantum dot (CQD) SCs [14,15], depleted heterojunction CQDSCs [16,17,18], quantum junction CQDSCs [19,20,21], and quantum dot-sensitized solar cells (QDSSCs) [22,23,24]. However, the PbS QDSCs that have been widely studied have only reached a record efficiency of 13.3% [25], which is far from the theoretical efficiency of 45% for a single-junction solar cell [26]. The main reason for this situation is that various sub-band gap trap states could be introduced in the synthesis and ligand exchange processes of CQD materials. CQDSCs are still limited by the trade-off between carrier extraction and light absorption. A typical planar n-p-p^+^-type PbS QDSC usually features an absorption layer that is only about 300 nm thick, while a thickness of QD film of about 1 μm is required to achieve the full absorption of solar radiation. Generally, limited carrier diffusion length (L_D_) and depletion width (W_D_) are the main factors limiting the thickness of the absorption layer [27]. Thus, maximizing the recombination lifetimes of both charge carriers and their transport properties, which are related to L_D_ and W_D_, is essential to achieve further advances in the thickness of the absorption layer. The IBHJ structure is considered to be another effective solution. IBHJ QDSCs feature a driving force to separate and transport the carriers with band bending and, at the same time, orthogonalize the direction of light absorption and electron–hole pair (EHP) separation, so that the absorber layer is thick enough to produce photocarriers that can be utilized [18]. Their n-type metal oxide acceptor is composed of 1D NWs (usually TiO_2_ or ZnO; here, we mainly focus on ZnO, since it easily synthesizes various nanostructures and offers higher electron mobility than TiO_2_). Simulation studies have shown that compared with planar solar cells, this type of cell structure is particularly advantageous when the optical absorption depth is comparable to the thickness of the device and the rate of carrier recombination in the depletion region is relatively large [28,29]. In addition, 3D structures have been shown to reduce surface optical reflection and enhance the absorption efficiency of SCs [30,31]. Moreover, the bending durability of ZnO NW arrays may offer promising prospects to meet the demands of flexible photovoltaic devices. Under bending conditions, interpenetrating structures can effectively release stress and thus resist the performance degradation caused by bending better than comparable planar devices [32]. Recently, a record short-circuit current density (JSC) of 33.2 mA cm^−2^ with a power conversion efficiency (PCE) of 10.62% was demonstrated for a PbS IBHJ QDSC employing the luminescence down-shifting method [33]. Further improvements of device performance require fine control of the morphology of NWs. For instance, in a piezoelectric strain sensor, inclined nanowires respond well to vertical contact forces, while vertically aligned nanowires are more sensitive to surface flow [34]. In IBHJ QDSCs, a better vertical arrangement of NWs can prevent the formation of large voids during the deposition of QDs [35]; the density, length, and surface defects of NWs directly affect the final performance of SCs. Besides, in real-world applications, different synthesis methods often feature different levels of morphology control precision, and the synthesis method must be considered to adapt to the application system of QDSC mass production. Therefore, it is necessary to review the preparation process of NW and analyze the morphology optimization mechanism of NWs.

In this study, several synthesis methods of ZnO NW are briefly introduced, ranging from vapor-phase methods and laser interference lithography to solution-based synthesis methods. The morphology control of hydrothermal-grown NW is mainly discussed, since hydrothermal (HT) method is simple, easy to operate, and suitable for large-scale application. Next, the effects of the geometrical morphology for the HT-grown ZnO NW on the performance of SCs is surveyed. Finally, the optimization strategies of NW morphology are discussed. Subsequently, the relationship between the process of photocarrier collection (electron transverse collection and hole longitudinal collection) and the morphology of NW is analyzed, and the control of the morphology of NW is proposed. In terms of interface optimization, the current strategies for the passivation of NW surface defects are summarized.

## 2. Preparation of ZnO Nanowires

Recently, ZnO NWs have gained considerable attention from the academic community for the fabrication of QD solar cells [36]. A variety of bottom-up approaches, including evaporation/chemical vapor deposition [37,38], solution-based synthesis methods (hydrothermal, electrodeposition) [39], and pulsed laser deposition (PLD) [40] has been exploited for the synthesis of ZnO NWs. Other growth methods, such as flame transport approach [41], electrospinning [42], molecular beam epitaxy (MBE) [43] etc., are also possible. Methods of controlling NW morphology by chemical vapor deposition and laser interference lithography are briefly introduced in the review, and then the hydrothermal method is mainly focused on, since it is simple and cheap.

### 2.1. Chemical Vapor Deposition

**Figure 1:**
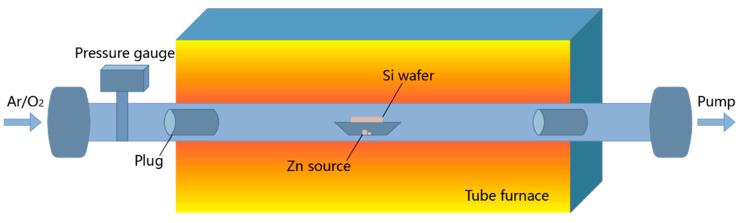
Schematic of CVD system used for the growth of ZnO NWs [44].

Typically, chemical vapor deposition (CVD) for the fabrication of ZnO NWs is carried out in a tubular furnace, as shown in Figure 1. The quartz boat with the Zn source and Si substrate were placed at the center of quartz tube, which was placed inside a tube furnace [44]. Generally, the formation of different diameters and lengths can be achieved by adjusting the flow rate of the carrier gas and the pressure during growth. In an experiment using argon as the carrier gas, the length and diameter of the NWs increase with the rise of oxygen supply. A further increase in the carrier gas flow rate triggers turbulent flow in the mixing process of the carrier gas and zinc vapor, and irregular results tend to occur [44]. Bhutto et al. [45] found that the final product was very short NWs, or even a layer of thin film at a low zinc vapor pressure, while the NWs grew with good orientation and uniform density at an appropriate vapor pressure. The condition of the substrate is another factor in the growth of aligned NW arrays. Well-aligned and uniform ZnO NW growth requires epitaxy, and the deposition of a c-oriented polycrystalline seed layer on a non-epitaxial substrate is an economical and versatile route [46]. Li et al. [47] found that the density of NWs increased and the degree of vertical arrangement improved with the increase in the seed layer thickness. Other studies have shown that the different deposition temperature and deposition methods of the ZnO seed layer also affect the orientation, morphology, and crystal quality of ZnO NWs grown by vapor deposition [47,48]. Furthermore, the metal catalysts in the vapor–solid–solid growth method are also important factors [49]. For example, neighboring metal catalysts may coalesce into larger-sized islands, leading to inferior control over the nanowire position and density when they are located at the semiconducting substrate during NW growth [50].

### 2.2. Laser Interference Lithography

**Figure 2:**
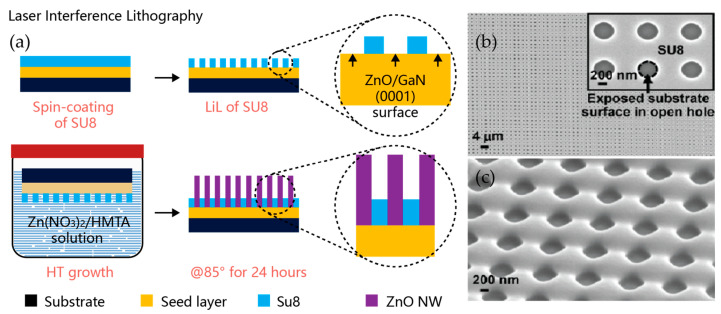
Schematics of the manufacturing process of ZnO NW arrays using laser interference techniques: (**a**) Fabrication process using the LIL approach for large-scale patterned ZnO NW arrays; (**b**) scanning electron microscope (SEM) image of patterned SU-8 film; (**c**) SEM image of patterned photoresist film [53].

The optical lithography and laser interference lithography (LIL) techniques, combining chemical and physical etching techniques to form patterned geometry, are more precisely designed and controllable methods used in the fabrication of ZnO nanostructures [51]. The LIL technique is based on the interference of two coherent light beams to form a horizontal standing wave pattern, which is applied to expose and record the patterns of periodic nanodot arrays (generally spin-coated onto a c-oriented ZnO seed layer) on the photoresist [52]. Wei et al. [53] demonstrated an approach to the patterned growth of ZnO NW arrays by combining the LIL and hydrothermal (HT) growth methods. The epoxy-based negative photoresist SU-8 was spin-coated onto a 2-inch silicon or sapphire wafer with a (0001) surface-oriented ZnO layer above it. A uniform pattern of open-hole arrays was formed at the unexposed locations of the SU-8 layer after two consecutive laser exposures. Next, the substrate with a patterned SU-8 layer was placed facing downward into the growth solution for the growth of ZnO NWs, as shown in Figure 2 [53]. In this way, the diameter, spacing, and length of the NWs can be well controlled.

### 2.3. Hydrothermal Method

Compared with other synthetic methods, the HT method has received widespread attention due to its advantages, including its low cost, low temperature, high yield, good expansibility, easy processing, and the flexibility it offers in the choice of both the synthesis pattern and the initial components (precursors and additives) [34,54,55]. The morphology and properties of ZnO NWs can be controlled by changing the growth parameters [56,57]. Generally, increasing the growth time causes the nanowires’ aspect ratio to increase gradually, which is attributed to the fact that the axial growth rate is higher than the lateral growth rate [58,59]. Muchuweni et al. [60] observed improved crystallinity and poorly aligned NWs with increasing growth time. The length and diameter of the ZnO NWs vary with the concentration of growth solution; a higher concentration yields a larger length and diameter [61]. Amin et al. [57] observed the change from NWs to nanopillars and finally polycrystalline thin films upon changing from lower concentrations (<25 mM) to higher concentrations (>400 mM). In a similar study, Gerbreders et al. [62] concluded that the minimum concentration value is 25 mM; concentrations lower than this would lead to incomplete nanorod formation with chaotic orientations since the solutions would be depleted too quickly. Consistent with the effect of solution concentration, higher temperatures enhance the driving force of the chemical reaction, giving rise to high-aspect-ratio NWs [58]. The ZnO NW is homogeneous and well-arranged under 90 °C, which is considered to be the optimal temperature in many articles [62]. The PH value is another important factor. Redundant ${\mathrm{OH}}^{-}$ ions suppress the influence of the competition mechanism, hence the difference in growth rate in the different directions [62,63]. When the pH value of the solution is reduced, the acidic environment may etch the seed layer and thus reduce the density of the NWs, while on the other hand, abundant Zn^2+^ results in the growth of ZnO NWs with larger lengths and diameters [56,57]. However, the growth of NWs is not observed at a low pH value (pH < 4.6 in the research of Amin et al. [57]). This behavior is probably attributable to the absence of hydroxide complexes ($\mathrm{Zn}{\left(\mathrm{OH}\right)}_{n}$), which are essential for the growth of ZnO NWs [64].

**Figure 3:**
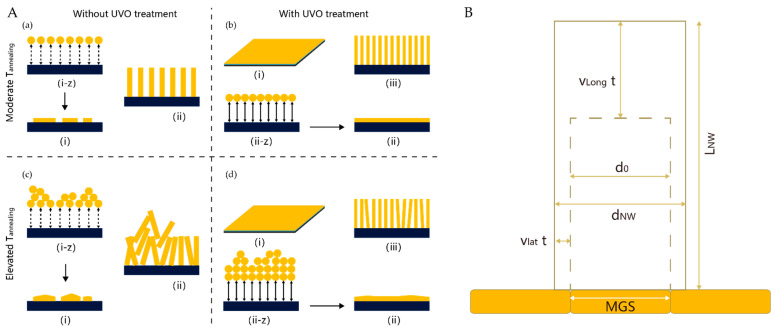
(**A**) Schematics of ZnO NW growth depending on substrate surface conditions and seed layer: (**a**) Without the ultraviolet ozone (UVO) treatment under a lower annealing temperature (T_annealing_); (**b**) with UVO treatment; (**c**,**d**) with varied T_annealing_ conditions [67]; (**B**) schematic representation of the NWs; growth [68].

As in the CVD method, substrate properties also play an important role. The effects of surface energy and seed layer annealing conditions on seed layer growth and HT-grown ZnO NWs were investigated by changing different conditions in the research of Park et al. [65], as shown in Figure 3a. Demes et al. [66] investigated the relationships between the characteristics of grown NWs and (i) the structural properties and morphologies of ZnO films (mean grain size (MGS), texture coefficient, and surface coverage rate) and (ii) growth duration time (t) in detail. The growth process of the NWs is shown in the Figure 3b. Specifically, the growth of the NWs starts from the nucleation site, with an initial diameter d0 at the seed layer surface, before proceeding along the lateral as well as the vertical direction at a rate of V_lat_ and V_long_, respectively. According to the authors’ observations, the main parameter influencing the NW aspect ratio and dimension appears to be the mean grain size of seed layers, while the texture coefficient of the seed layer can greatly influence the NW density [66]. In addition, the morphological properties of NWs grown using the HT method can also be optimized by postprocessing. Here, we briefly summarize the dominant factors affecting the morphology of hydrothermally grown NWs (see Table 1).

## 3. Effect of ZnO Nanowire Morphology on the SCs

### 3.1. Basic Physical Characteristics for IBHJ Photovoltaic Device

**Figure 4:**
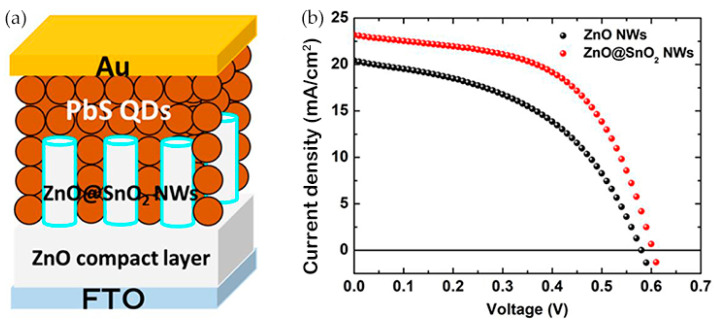
(**a**) Structural illustration of ZnO NW-based CQDSCs with SnO_2_ passivation layers; (**b**) the J-V characteristics of ZnO NW CQDSCs with or without SnO_2_ passivation [92].

The current density in typical silicon p–n junction SCs is given by the sum of the drift currents in an electric field and the diffusion currents [79,80],

(1) $$J=\mathrm{qE}\left({n\mu }_{e}+{p\mu }_{h}\right)+q\left(D_{e}\frac{\mathrm{dn}}{\mathrm{dx}}+D_{h}\frac{\mathrm{dp}}{\mathrm{dx}}\right)$$

where n and p are the electron and hole concentration, respectively, μ_e(h)_ is the majority carrier mobility, E is the electric field in the device, and D_e_ and D_h_ are the electron and hole diffusion coefficients. Generally, the CQD absorption layer is treated as a bulk semiconductor to describe phenomena such as drift and diffusion, carrier generation and recombination, and transport and trapping in CQD films [81]. In a depleted heterojunction CQDSC, a planar heterojunction is formed between the QD film and a wider bandgap electrode, which establishes a depletion region. The effective carrier extraction length is defined as W_QD_ + L_D_, where W_QD_ is the width of the depletion region residing in the QD absorption layer and L_D_ is the minority carrier diffusion length [82]. The depletion region width is determined by the doping concentration of the material, which features a trade-off with the open circuit voltage (V_OC_). QD materials with low defect density and high carrier mobility enable the efficient extraction of photogenerated carriers in the quasineutral region through the diffusion process. Photocarriers generated in regions further away from the depletion region than their L_D_ recombine before they can be extracted. This limited extraction length results in a compromise between the extraction of carriers and the absorption of light. IBHJ structures can break this balance by decoupling the directions of photon absorption and charge collection. A typical IBHJ structure is shown in Figure 4a, where a three-dimensional depletion heterojunction is formed between the NW and the QD absorber layer. Due to the optimized collection of optical carriers and improved light trapping effect, QDSCs employing this structure usually feature enhanced short-circuit current density (J_SC_) than their planar counterparts. The PV performances of the QDSCs incorporating ZnO NWs are gathered in Table 2.

The depletion width of the CQD photovoltaic devices can be characterized by employing capacitance-voltage spectroscopy. The total depletion width (W_DT_) of the device can be obtained as follows [94],

(2) $$W_{\mathrm{DT}}=\sqrt{\frac{{\mathrm{qN}}_{\mathrm{QD}}N_{\mathrm{ZnO}}\varepsilon_{\mathrm{QD}}\varepsilon_{\mathrm{ZnO}}}{2\left(\varepsilon_{\mathrm{QD}}N_{\mathrm{QD}}+\varepsilon_{\mathrm{ZnO}}N_{\mathrm{ZnO}}\right)}\left(V_{\mathrm{bi}}-V\right)}$$

where N_i_ and ε_i_ (i = QD, ZnO) are the carrier density and permittivity of the n-type ZnO and p-type QDs, respectively. The value V_bi_ is the built-in potential and V is the applied bias. N_ZnO_ is usually the known parameter while, the carrier concentration of the CQD film is always obtained using Mott–Schottky analysis by fitting the linear region of 1/C^2^ (vicinity of V_bi_). From W_DT_, the corresponding depletion region into the CQD film can be obtained as

(3) $$W_{\mathrm{QD}}=W_{\mathrm{DT}}\frac{\varepsilon_{\mathrm{ZnO}}N_{\mathrm{ZnO}}}{\varepsilon_{\mathrm{ZnO}}N_{\mathrm{ZnO}}+\varepsilon_{\mathrm{QD}}N_{\mathrm{QD}}}$$

With the lifetime and mobility known (mobility can be calculated from the SCLC regime), the diffusion length can easily be calculated according to the formula,

(4) $$L_{D}=\sqrt{\frac{k_{B}T}{e}\mu \tau }$$

where $k_{B}$ is the Boltzmann constant, T is the temperature, e is the elementary charge, µ is the electron mobility, and τ is the carrier lifetime. Other measurement methods of L_D_ can be seen in the study of Zhitomirsky et al. [95].

### 3.2. Density of Nanowires

Generally speaking, the density of NW arrays is an important factor affecting the performance of IBHJ SCs. Although NW arrays with high density can speed up the extraction of photocarriers, this leads to a lower volume filling fraction of the QD light-absorbing materials. More importantly, NW arrays with high density feature larger interface areas, which undoubtedly increase the carrier recombination at the interface and, hence, a dramatic V_OC_ loss. In addition, studies have shown that appropriate density control can enhance the capture ability of incident light. Therefore, it is of great practical significance to analyze the density regulation mechanism of NW arrays in detail. Progress in this area has generally been slow for two reasons. On the one hand, accurate and high-quality synthesis methods, such as photolithography and vapor deposition, often require expensive equipment, which runs counter to the goal of the large-scale application of QD photovoltaic devices. On the other hand, NWs grown by simple hydrothermal synthesis methods usually feature higher area density and more surface defects, and their morphology control is usually more complex.

**Figure 5:**
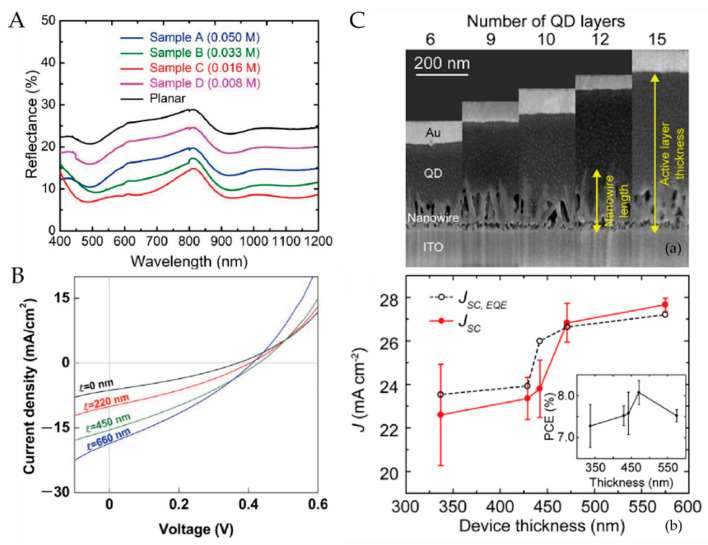
(**A**) Reflectance spectra of ZnO NWs with different areal densities grown on certain substrate [93]; (**B**) I-V curves of CQDSCs assembled with different length NWs [83]; (**C**) (**a**) SEM images of NW CQDSCs with different NW lengths, (**b**) corresponding device performance of different CQD layers [91].

A typical IBHJ SC prepared using hydrothermal NW array is shown in Figure 8a. Due to the high density of the NWs, the QD materials between NWs are usually completely exhausted. In this case, the surface recombination of NWs and the light harvesting efficiency (LHE) are the main considerations. Theoretically, the lower the nanowire density, the less constrained the surface recombination, provided that carriers can be collected efficiently. The usefulness of surface defect passivation is a clear indirect proof of this point. Ozu et al. [92] used ${\mathrm{SnO}}_{2}$ layers to passivate the surface of ZnO NWs, and the PCE of the NW CQDSCs was improved from 5.6 to 7.8% (Figure 4). They attributed this to the reduced deep-level defect density and an optimized band-gap arrangement. For effective light management in the IBHJ structures, too large or too small an area density leads to higher light reflectance, as shown in Figure 5a, and an appropriate area density eventually leads to higher light absorption and higher J_SC_. Recently, Tavakoli Dastjerdi et al. [93] obtained a champion device with a PCE of 10.1% by tuning the areal density of ZnO NWs (≈150 per μm^2^) by controlling the precursor concentration. In short, the density control of NWs requires a balance between the interface area and the light trapping effect of NWs. However, this is all based on satisfying the effective carrier collection, because spacing that is too large leads to poor carrier collection between NWs.

### 3.3. Length of Nanowires

The length of NWs is another important factor affecting device performance, and related studies have been widely reported. Studies have shown that both the total PCE and J_SC_ increase with the length of NWs under the condition of constant thickness of the QD absorption layer, as revealed in Figure 5b [83]. The PCE of the device increases with the thickness of the QD layer and eventually decreases due to bulk recombination and increasing series resistance under the condition that the length of NWs is constant as shown in Figure 5c [91]. In principle, long NW arrays allow the device to adopt a thicker QD absorption layer and can also enhance the light harvesting efficiency (LHE). However, increased device thickness eventually results in the deterioration of the carrier collection for a constant photocarrier diffusion length. Increasing the nanowire length causes poorer charge collection, since holes photoexcited near the front electrode must travel a long distance [88]. In addition, longer NWs mean a larger surface area, which leads to an increase in interfacial recombination events and, thus, a decreased V_OC_.

**Figure 6:**
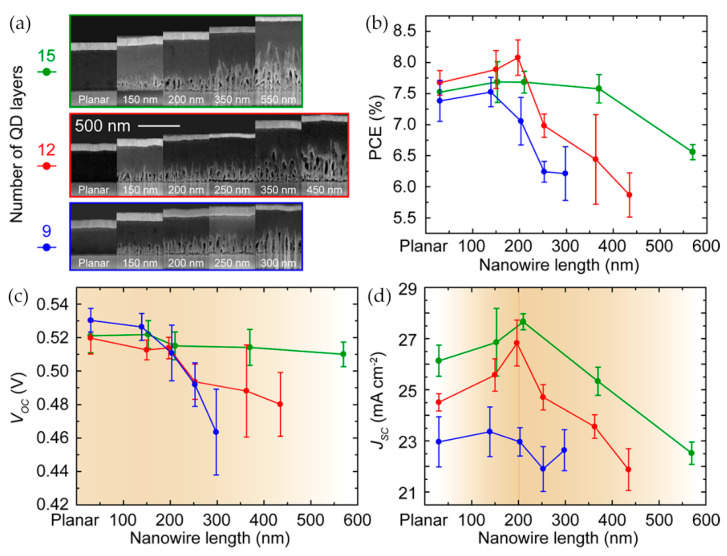
Performance variations of devices with different characteristics: (**a**) SEM images of devices with different NW lengths and CQD layers; (**b**) PCE, (**c**) V_OC_, and (**d**) J_SC_ for sets of devices with different conditions [91].

Recently, Cheng et al. [91] fabricated devices with varied NW lengths to investigate the role of NWs in SCs. They found that the PCE reached its maximum under certain conditions and then gradually decreased, as shown in Figure 6. Through frequency domain time difference (FDTD) simulation, they found that internal light scattering and local light concentration caused by 3D structures may result in changes to carrier recombination dynamics. Increased internal scattering with longer NWs causes the light to concentrate in a certain region and, hence, an increased carrier recombination rate. Therefore, the light trapping effect of NWs needs to be studied in detail to evaluate whether they are beneficial or harmful to a particular device. Notably, the QD absorber layer must slightly cover the NW arrays to avoid direct contact between the top ohmic contact and the ZnO. Jean et al. [84] found that the thermally evaporated MoO_3_ interlayer, used to protect the QDs from damage during the destructive reverse contact deposition, could also act as a physical buffer layer to reduce the problem of short-circuit between the NWs and the back electrode.

## 4. Optimization Strategies of IBHJ QDSCs Based on ZnO NWs

### 4.1. Geometrical Morphology Optimization of Nanowires

**Figure 7:**
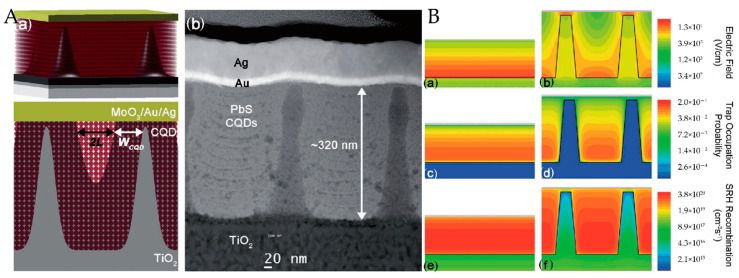
(**A**) (**a**) Schematic and (**b**) SEM images of CQDSCs; (**B**) electric field distributions, trap occupation, and SRH recombination of planar and nanopillar architectures [96].

The original intention to develop this bulk heterojunction structure was to increase the electrical contact area between the donor and the acceptor so that the photocarriers can be collected before they are captured by trap states. Moreover, NWs allow the light absorption and carrier extraction to be controlled independently [83]. Therefore, our core aim can be summarized as obtaining efficient carrier extraction and the increased thickness of light-absorbing materials. As early as 2012, Kramer et al. [96] proposed the spacing of nanopillars in their research on TiO_2_ NWs QDSCs (Figure 7). However, longitudinal carrier collection is neglected, and this process often affects the overall thickness of the SCs.

**Figure 8:**
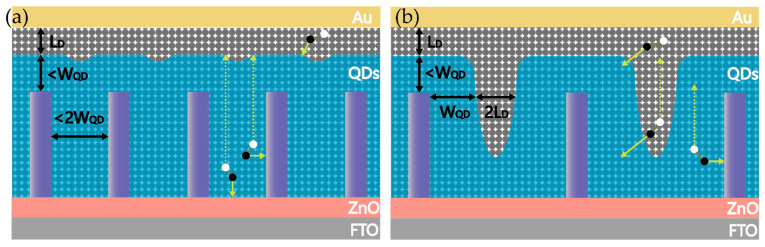
(**a**) IBHJ structure based on hydrothermal method; (**b**) ideal IBHJ structure.

Considering the efficient collection of photocarriers, it is necessary to distinguish between the electron and hole collection processes. To collect photogenerated electrons efficiently, the pitch size of the NWs should meet the following requirements (Figure 8),

(5) $$d_{\mathrm{np}-\mathrm{np}}<2\times \left(W_{\mathrm{QD}}+L_{D}\right)$$

(6) $$d_{\mathrm{top}}<W_{\mathrm{QD}}+L_{D}$$

where $d_{\mathrm{np}-\mathrm{np}}$ is the average spacing of NWs and $d_{\mathrm{top}}$ is the distance from the top of the NW to the back electrode. Notably, $d_{\mathrm{top}}$ is an upper bound on collection length. The depletion situation at the top of the nanowires is usually more complicated. Electrons are mainly collected horizontally, whereas holes often need to be transported vertically to the host electrode to form photocurrents. Therefore, the limited collection length of holes directly limits the length of the NWs and, thus, the thickness of the light-absorbing layer material,

(7) $$D_{\mathrm{QD}}<L+W_{\mathrm{QD}}+L_{D}$$

where $D_{\mathrm{QD}}$ is the thickness of the QD absorption layer and L is the mean length of the NWs, which is limited to the hole collection length. Although studies have shown that holes in PbS QDs can diffuse a distance of over 1000 nm or longer under certain conditions [88], experimental and theoretical analysis is still needed to determine the optimal hole collection length. There are indications that even if IBHJ QDSCs with ultra-long NWs (1 μm to 2 μm) offer considerable efficiency, poor hole collection is likely to be the main factor limiting their efficiency. Experiments have shown that the external quantum efficiency decreases as the NWs grow in the short-wavelength regions, while the near-infrared band light increases as the NWs grow; this seems to be a good proof [91], since short-wavelength light is usually absorbed at the top of the cell and the holes usually feature a long collection distance. A QD absorption layer in the NW root area is likely to form a dead zone because holes are likely to quench or be captured in traps in the process of diffusion to the back electrode. Only near the NW top and the bottom of the SCs do the light absorption layer, NWs, and back electrode form an effective electrical pathway. A more detailed mechanism can be explored to a certain extent by using more sophisticated FDTD modeling to simulate the recombination of carriers in the QD region between the NWs. Furthermore, it is worth mentioning that the above parameters usually need to be determined according to the actual situation. For example, the width of the depletion region on the QD side usually depends on the doping concentration of the material. Here, we summarize some parameters of the bulk heterojunction formed between PbS quantum dots passivated by different ligands and ZnO in recent years (Table 3).

As the main geometric parameter, the diameter of the nanowires has rarely been mentioned. Generally, the diameter of the NWs should not be too small, despite their high doping relative to QD materials. To achieve the maximum depletion width in the radial direction of the NWs, the diameter of the nanowire must be at least twice the depletion width of the ZnO NW [29]. Moreover, NWs that are too thin are easy to break during assembly due to their poor mechanical properties. In order to satisfy these conditions, the light trapping effect should receive sufficient attention. A high LHE can be achieved by adjusting the density and length of the NWs. This process, however, is often associated with the surface area of the nanowires. Striking a balance between low surface area and optimized light capture is also a critical issue. The final consideration is the surface passivation of the NWs, which is discussed in the next section.

### 4.2. Passivation of Defects in Nanowires

**Figure 9:**
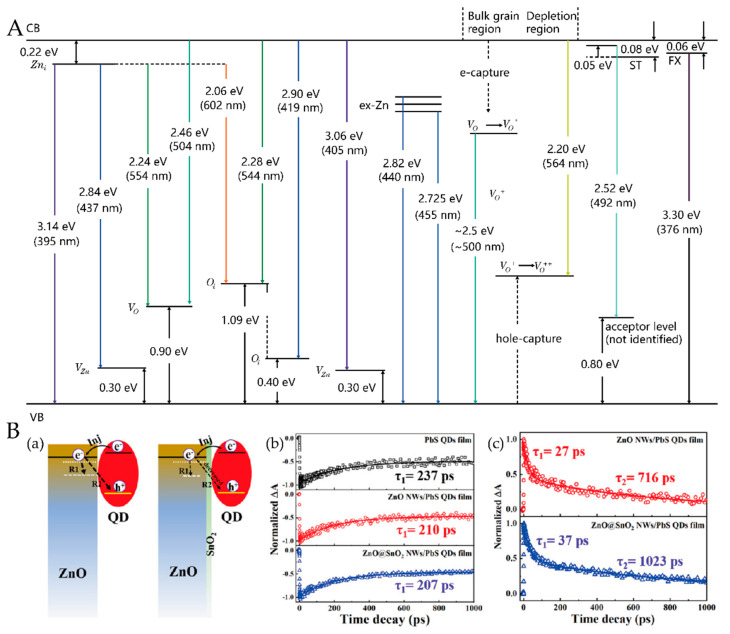
(**A**) Energy level diagram showing some of the principal defect levels in ZnO [101]; (**B**) (**a**) charge dynamic processes occurring at the ZnO NW/QD film interface. TA decay curves of the PbS QD film, ZnO NW/PbS QD film, and ZnO@SnO_2_ NW/PbS QD film with a pump light of 470 nm and probe lights of (**b**) 960 and (**c**) 1500 nm [104].

ZnO NWs prepared using the low-temperature HT procedure usually feature more defect states, including shallow-level trapping states originating in the vacancies or interstitials of zinc [101]; and deep level trapping states, which are formed by oxygen vacancy, hydroxyl groups, and excess oxygen [102]. Studies have shown that shallow defect states in ZnO can not only improve the electron mobility of ZnO, but also act as an additional path for electron injection from the QD layer to the NWs in some cases [103]. Instead, the deep-level trapping states are the critical target of defect passivation because they mainly serve as the recombination centers of photocarriers [104]. Generally, green and yellow emissions in the PL spectra of ZnO are assigned to deep-level defects (red emissions are associated with either surface oxygen or surface OH groups), while violet and blue emissions are related to shallow defects [101]. A relevant description is presented in the article by Vempati et al., as shown in Figure 9a. Hence, we can estimate the defect density of ZnO NWs from these emissions.

The defect concentrations of HT-grown ZnO NWs can be significantly reduced by annealing [105]. Xu et al. [106] successfully eliminated or passivated the defect-related PL emissions of ZnO NWs by employing sealed post-annealing treatment. Concerning again the surface defects of NWs, Kim et al. [107] successfully reduced the number of excess-oxygen-related defects through thermal annealing in an $H_{2}$ ambient atmosphere above 300 ℃. The excess-oxygen-related deep-level emissions were quenched completely, while the near band-edge emissions were greatly enhanced when H atoms were incorporated into ZnO NWs. These H atoms are most likely to exist in the form of shallow donors and may be removed after annealing at 400 ℃ [108,109,110]. Concerning the desorption of hydroxyl groups, Shi et al. [108] confirmed that annealing at 150 ℃ results in the removal of OH groups, but it may also introduce other hydrogen-related impurities. In a study of the overall performance of devices, Chang et al. [86] found that 350 ℃ might be the optimal annealing temperature, and the lowest defect emission and sufficient ZnO crystallinity were obtained at this temperature.

More recently, Tavakoli Dastjerdi et al. [93] passivated ZnO NW surface states using the hydrogen plasma treatment. Compared with the untreated NWs, the PL spectrum of the H-plasma-treated ZnO NWs showed a clear enhancement of near-band emissions and a remarkable quenching of the visible emission peak. Through X-ray electron spectroscopy (XPS) analysis, the authors found an increase in the number of hydroxyl groups and attributed this to the passivated O-dangling bonds. Despite the increase in hydroxyl groups, reduced surface oxygen defects and possible H doping may lead to an improved overall performance [93,107]. Shi et al. [35] obtained a 9.3% PCE by introducing ABA-modified ZnO NW arrays in QDSCs. In addition to the passivation of ZnO surface defect states, ABA modification can change the interfacial dipole momentum between ZnO NWs and CQDs, thus effectively increasing the vacuum level.

Parallel to the class of passivation strategies for post-annealing and surface treatment, depositing organic/inorganic coatings to passivate the surface of ZnO NWs were also investigated [91,109]. Zhang et al. [104] clarified in detail the influence of surface ${\mathrm{SnO}}_{2}$ passivation on the charge dynamic process in ZnO NWs by employing time-resolved photoluminescence (TRPL) and transient absorption (TA) spectroscopy techniques, as shown in Figure 9b. After ${\mathrm{SnO}}_{2}$ passivation, the PL intensity of the deep-level defects related emission clearly decreased and the time constants of the two recombination pathways (R1 pathway, in which free electrons are trapped by defect states, and the R2 pathway, in which free electrons are recombined with the holes of PbS QDs) increased, which confirmed the positive effect of the surface passivation layer. Interestingly, Zang et al. [89] revealed the possibility of insulating interfacial modification with a Mg(OH)_2_ interlayer; the performance of the IBHJ QDSCs improved, with a V_OC_ and PCE of 520 mV and 5.04%, respectively. Through the chemical reaction between ZnO and the TiO_2_ precursor, Chang et al. [86] fabricated ${\mathrm{TiO}}_{2}$-passivated ZnO NW CQDSCs. They provided direct proof of the reduced recombination in the surface-passivated cell by transient open-circuit photovoltage decay measurements. The results showed that the ${\mathrm{TiO}}_{2}$-passivated cells exhibited much slower decay processes than those of the bare unpassivated cells. More recently, Cheng et al. [91] significantly minimized $V_{\mathrm{OC}}$ losses by passivating a ZnO nanowire surface with ALD ${\mathrm{TiO}}_{2}$. Compared with unpassivated devices, the surface-passivated NW devices featured higher $J_{\mathrm{sc}}$ and $V_{\mathrm{OC}}$, which was attributed to the passivated surface defects. The performance comparison before and after the passivation process can be seen intuitively in Table 4.

### 4.3. Other Optimization Strategies

The effective infiltration of QDs in NW arrays is an important means of reducing interfacial defects. However, an important fact is that IBHJ QDSCs may feature a large number of voids, and these voids could cause a sharp decline in the performance of devices. Recently, more and more evidence has shown that the deposition method exerts an important effect on the morphology of thin films. Although the traditional spin-coating deposition method features advantages in small-scale fabrication due to its simplicity and practicability, it may be difficult to control the morphology due to the uneven shear stress distribution during the coating process [86]. In addition, it is still debatable whether mechanical fractures occur in the process of high-speed rotation of NW arrays, which aggravate the deterioration of the performance. A new deposition method based on the meniscus was proposed. “Meniscus-guided” means that the meniscus is shifted across the substrate by a coating head or viscous force to effectively guide and control film deposition [110]. Due to the inherent directivity of the coating process, the QDs can be effectively deposited by penetration. This method is appropriate for large-scale use. By simultaneously controlling the growth orientation of the NWs and introducing convective assembly as the CQD deposition technique, the dense packing and efficient infiltration of CQDs can be effectively improved. In this way, Shi et al. produced a finely interpenetrating OBHJ structure between ZnO NW arrays and PbS CQDs, and demonstrated a PCE of 9.92% in the devices [35].

QDSC design for stability is also an important subject for PV commercialization. Without considering the NW light aggregation effect and similar structural issues, IBHJ QDSCs are similar to planar QDSCs in that their device lifetime is mainly dependent on the QD material itself. Current synthesis and defect passivation techniques have been effective in reducing the oxidation sites associated with the stability of materials. PbS QDSCs can maintain their initial PCE in air with no encapsulation for 30 days [108]. In another study, ZnO NW-based CQDSCs exhibited excellent stable properties (the PCE was almost constant) after being stored in air for 250 days [92]. Further stability optimization strategies need to consider the mechanism of ambient factors (such as oxygen, water, and increased temperatures or high intensities of illumination) affecting the QDs. The intrinsic properties of the QDs themselves and their interplay with other materials are also factors to be considered [110].

## 5. Conclusions

IBHJ QDSCs form a 3D depletion region for the high-efficiency utilization of solar energy, which allows the incorporation of more QD material while maintaining efficient charge collection. As ETLs, ZnO NW structures should offer optimal electric continuity and need to be relatively transparent to visible light to avoid photon loss. In this study, an assessment was performed on the previous research into the preparation of ZnO NWs, and the effect of the ZnO NW morphology on performance and the optimization strategies of IBHJ QDSCs were summarized. The conclusions and outlooks are as follows:

(1) The HT method is more suitable for the large-scale application of IBHJ QDSCs due to its advantages of low temperature, high yield, and easy processing. The morphology of HT-grown NWs is influenced by the seed layer properties, growth time, concentration, temperatures, PH, and post-treatment conditions. However, it is necessary to investigate the mechanism of these influencing factors for the preparation of NWs with controllable morphologies. Furthermore, the optimization of surface defects and internal defects and the reliability of HT-doping elements also need to be studied further to improve the performance of the ETL and to tune the band-gap arrangement.
(2) The density of the NW affects the filling volume of QD materials. High-density NW arrays not only feature a large V_OC_ loss due to their large interface area, but also cause high reflectivity in the SCs, reducing the light absorption. The length of the nanowires determines the depth of the carrier collection and further affects the thickness of the SCs. However, long NWs often result in poor hole collection and increase the interface area. In addition, the length of the nanowires affects the light trapping effect of the 3D structure, and may cause the aggregation of light absorption and ultimately affect the recombination of the carriers.
(3) The geometric morphology of the NW exerts a great influence on the transmission and collection of photocarriers. The optimization of NW spacing needs to take account of the transverse collection of electrons, while the length of the NW and the thickness of the SC absorption layer need to be determined after comprehensively analyzing the longitudinal aggregation of holes. In order to satisfy the carrier collection conditions as far as possible, reducing the NW density can increase the filling volume of QD light absorption material.
(4) In order to further optimize the 3D structure, more accurate carrier transport mechanism analysis needs to be carried out in combination with electrical simulation to determine the optimal electrical structure. The light-trapping effect caused by NWs also needs to be more accurately analyzed, since poor light scattering may lead to local light aggregation and thus affect the distribution and collection efficiency of photocarriers.
(5) The passivation of the NW surface is an important way to improve the V_OC_ of IBHJ QDSCs. Passivation strategies commonly include post-annealing, H-plasma treatment, and ABA surface treatment. Moreover, the addition of Mg(OH)_2_, SnO_2_, and TiO_2_ buffer layers on the surface of NWs also effectively improves the performance of SCs. However, the effects of the passivation layer (including the passivation mechanism of the defects and the band arrangement of the battery) on the three-dimensional heterojunction remain to be explored. A comparative study of different passivation layers may be helpful in this regard.

## Figures and Tables

**Table 1:** Factors influencing the morphology of hydrothermally grown NWs.

Conditions		Verticality	Density	Mean Length	Mean Diameter	Crystallinity
Seed layer properties	Thickness [67,68,69]	-	√	-	√	-
	Surface roughness [65,68,69]	√	√	-	√	-
	Texture coefficient [66]	-	√	-	-	-
	Mean grain size [66,70]	-	√	√	√	-
	Nucleation site density [69,71]	-	√	-	-	-
	Crystal orientation [67,72]	√	-	-	-	√
Growth time [57,60,62,73]		-	-	√	√	√
Concentration of solution [57,60,62,74]		-	√	√	√	√
Temperatures [57,60,62,73,75]		√	-	√	√	√
pH [57,62,75,76,77]		-	√	√	√	-
Post-annealing treatment [77,78]		√	-	-	-	√

**Table 2:** PV performances of the QDSCs integrating ZnO NW arrays.

Photoanode	QD Material	IBHJ QDSC Structure	J_SC_ (mA cm^−2^)	V_OC_ (mV)	FF	PCE (%)
ZnO NWs	PbSe [83]	ITO/ZnO/PbSe-EDT/α-NPD/Au	18.6	420	0.25	1.97
	PbS [84]	ITO/ZnO/PbS-BDT/MoO_3_/Au	17.9	600	0.40	4.3
	PbS [85]	FTO/ZnO/PbS-CTAB/Au	34.47	361	0.488	6.074
	PbS [86] ^a^	FTO/ZnO/PbS-CTAB/Au	30.7	420	0.478	6.16
	PbS [87]	FTO/ZnO/PbS-CTAB/Au	27.9	422	-	6.03
	PbS [88]	FTO/ZnO/PbS-CTAB/Au	28.8	419	0.47	5.7
	PbS [89] ^b^	FTO/ZnO/PbS-TBAI/PbS-EDT/Au	21.51	520	0.450	5.04
	PbS [36]	ITO/ZnO/PbS-TBAI/PbS-EDT/Au	29.4	570	0.57	9.6
	PbS [32] ^c^	ITO/ZnO/PbS-TBAI/PbS-EDT/Au	17.81	490	0.435	3.81
	PbS [90]	FTO/ZnO/PbS-TBAI/Au	27.6	351	0.486	4.70
	PbS [91] ^d^	FTO/ZnO/PbS-TBAI/PbS-EDT/Au	26.0	497	0.56	7.2
	PbS [92] ^e^	FTO/ZnO/PbS-CTAB/Au	23.2	603	0.56	7.78
	PbS [35] ^f^	ITO/ZnO/PbS-TBAI/PbS-EDT/Au	27.5	540	0.64	9.52
	PbS [93] ^g^	ITO/ZnO/PbS-TBAI/PbS-EDT/Au	31.1	610	0.57	10.8

^a,d^ TiO_2_-passivation. ^b^ Mg(OH)_2_-passivation. ^c^ Flexible IBHJQDSC. ^e^ SnO_2_-passivation. ^f^ ABA(4-aminobenzoic acid)-passivation. ^g^ H-plasma-passivation.

**Table 3:** Characteristic parameters of several lead chalcogenide QD material layers.

Ligand	CQD Material	CQDSC Structure	Doping Density (cm^−3^)	Depletion Width (nm)	Diffusion Lengths (nm)	PCE (%)
Organic ligand	PbS-MPA [95]	-	-	-	30	-
	PbS-MPA [97]	-	-	-	70	-
Halide ligand	PbS-PbX_2_ [98]	ITO/ZnO/PbS-PbX_2_/PbS-EDT/Au	9.94 × 10^16^	165	52	7.2
	PbO-PbS-TBAI [99]	ITO/ZnO/PbS-TBAI/PbS-EDT/Au	-	-	61	9.4
	PbAc-PbS-TBAI [99]	ITO/ZnO/PbS-TBAI/PbS-EDT/Au	-	-	95	10.8
	PbS-TBAI [95]	-	-	-	70	-
	PbS-TBAI [94]	ITO/ZnO/PbS-TBAI/PbS-EDT/Au	7.3 × 10^16^	61.0 (at maximum PCE)	85	8.7
	PbS-I_2_ + TBAI [94]	ITO/ZnO/PbS-I_2_ + TBAI/PbS-EDT/Au	6.8 × 10^16^	62.5 (at maximum PCE)	115	10.1
Hybrid ligand	PbS-MPA + CdCl_2_ [100]	FTO/ZnO/PbS-MPA +CdCl_2_/MoO_x_/Au/Ag	1 × 10^15^–1 × 10^16^	-	80	7.6
	PbS-PbX_2_ + MPE [98]	ITO/ZnO/PbS-PbX_2_ + MPE/PbS-EDT/Au	1.96 × 10^16^	288	94	9.6
	PbS-MPA/CdCl_2_ [97]	-	-	-	230	-

**Table 4:** Performance optimization of IBHJ QDSCs with passivation strategies.

Passivation Strategy	CQDSC Structure	-	J_sc_ (mA/cm^2^)	V_oc_ (mV)	FF	PCE (%)
H-plasma-passivation [93]	ITO/ZnO NWs/PbS-TBAI/PbS-EDT/Au	non-treated	30.0	591	0.56	9.9
		H-plasma treated	31.1	610	0.57	10.8
ABA-passivation [35]	ITO/ZnO NWs/PbS-TBAI/PbS-EDT/Au	non-treated	27.4	510	0.60	8.41
		ABA-treated	27.5	540	0.64	9.52
Mg(OH)_2_ [89]	FTO/ZnO NWs/PbS-TBAI/PbS-EDT/Au	without Mg(OH)_2_	22.62	390	45.66	4.03
		with Mg(OH)_2_	21.51	520	45.08	5.04
SnO_2_ [92]	FTO/ZnO NWs/PbS-CTAB/Au	without SnO_2_	20.4	580	0.47	5.55
		with SnO_2_	23.2	603	0.56	7.78
CBD-TiO_2_ [86]	FTO/ZnO NWs/PbS-CTAB/Au	without TiO_2_	28.7	279	0.430	3.50
		with TiO_2_	30.7	420	0.478	6.16
ALD-TiO_2_ [91]	FTO/ZnO NWs/PbS-TBAI/PbS-EDT/Au	without TiO_2_	25.3	505	0.54	6.9
		with TiO_2_	26.0	497	0.56	7.2
